# Mapping the Posterior Ledge and Optic Foramen in Orbital Floor Blowout Fractures

**DOI:** 10.1055/a-2074-2092

**Published:** 2023-08-02

**Authors:** Yu Cong Wong, Doreen S.L. Goh, Celine S.Y. Yoong, Cowan Ho, Elijah Z. Cai, Angela Hing, Hanjing Lee, Vigneswaran Nallathamby, Yan L. Yap, Jane Lim, Sundar Gangadhara, Thiam C. Lim

**Affiliations:** 1 Department of Surgery, Yong Loo Lin School of Medicine, National University of Singapore, Singapore; 2 Department of Surgery, Division of Plastic, Reconstructive and Aesthetic Surgery, National University Health Systems, Singapore; 3 Department of Surgery, Yong Loo Lin School of Medicine, National University Health Systems, Singapore; 4 Department of Surgery, Division of Plastic, Reconstructive and Aesthetic Surgery, Ng Teng Fong General Hospital, Singapore; 5 Department of Ophthalmology, Division of Orbit and Oculofacial Surgery, National University Hospital, Singapore

**Keywords:** orbital fractures, blowout fractures, optic nerve

## Abstract

**Background** The posterior ledge (PL) is a vital structure that supports the implant posteriorly during orbital floor reconstruction. This study describes a technique for mapping the PL in relation to the infraorbital margin (IM) in patients with orbital floor blowout fractures. This study establishes the location of the optic foramen in relation to the PL.

**Methods** Facial computed tomography (FCT) scans of 67 consecutive patients with isolated orbital floor blowout fractures were analyzed using Osirix. Planes of reference for orbital fractures, a standardized technique for performing measurements on FCT, was used. Viewed coronally, the orbit was divided into seven equal sagittal slices (L1 laterally to L7 medially) with reference to the midorbital plane. The distances of PL from IM and location of optic foramen were determined.

**Results** The greatest distance to PL is found at L5 (median: 30.1 mm, range: 13.5–37.1 mm). The median and ranges for each slice are as follows: L1 (median: 0.0 mm, range: 0.0–19.9 mm), L2 (median: 0.0 mm, range: 0.0–21.5 mm), L3 (median: 15.8 mm, range: 0.0–31.7 mm), L4 (median: 26.1 mm, range: 0.0–34.0 mm), L5 (median: 30.1 mm, range: 13.5–37.1 mm), L6 (median: 29.0 mm, range: 0.0–36.3 mm), L7 (median: 20.8 mm, range: 0.0–39.2 mm). The median distance of the optic foramen from IM is 43.7 mm (range: 37.0– 49.1) at L7.

**Conclusion** Distance to PL from IM increases medially until the L5 before decreasing. A reference map of the PL in relation to the IM and optic foramen is generated. The optic foramen is located in close proximity to the PL at the medial orbital floor. This aids in preoperative planning and intraoperative dissection.

## Introduction

Orbital floor reconstruction involves the placement of a reconstruction plate over the infraorbital margin (IM) and posterior ledge (PL) to restore contour and volume. 1 Computed tomography (CT) remains the gold standard for identifying and characterizing orbital fractures. 2 3 A reference map of the defect will assist surgeons in dissection and choosing appropriately sized implants. The optic foramen is a critical structure to avoid in orbital dissection, and it is difficult to delineate intraoperatively. 4 Trauma to the optic nerve during surgery causes blindness. 5 This study presents a reproducible technique for mapping the outline of PL and assessing the location of the optic foramen in relation to PL and IM.

## Methods

A total of 278 consecutive patients with orbital floor fractures who sought treatment from 2002 to 2015 were recruited for the study. Patients with bilateral orbital floor fractures and those with fractured IM were excluded. Sixty-seven patients with unilateral isolated orbital floor blowout were eligible for this study.

CT scans were analyzed using Osirix Digital Imaging and Communications in Medicine Viewer v9.0.1 (Pixmeo SARL, Geneva, Switzerland).

Measurements were made using the following window width (ww) and window level (wl) settings:

● Bone window: ww: 1,500  Hounsfield unit (HU); wl: 300 HU 6
● Brain window: ww: 100 HU; wl: 50 HU 7
Bone window was the default window setting for all measurements except when obtaining the retroglobe plane (brain window was used).

### Procedure

**Fig. 1:**
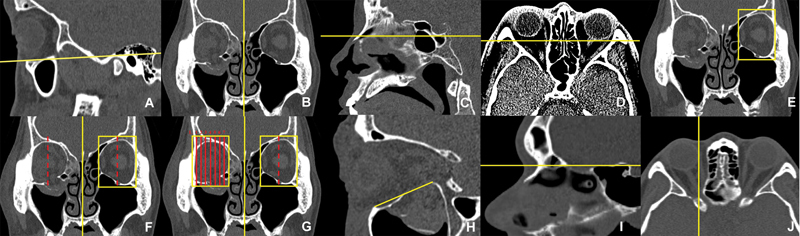
Frankfurt plane ( **A** ), midsagittal plane ( **B** ), lowest point of sella turcica ( **C** ), retroglobe plane ( **D** ), medial and lateral most points of unfractured orbit ( **E** ), midpoint of fractured orbit/L4 ( **F** ), L1–7 ( **G** ), distance between infraorbital margin and posterior ledge ( **H** ), change in angle between superior edge of sella turcica and base of cranial fossa ( **I** ), vertical line intersecting with anterior–superior most point of sphenoid bone forming the optic foramen ( **J** ). All figures are in the bone window except ( **D** ; brain window). Measurements were all made using the following Hounsfield unit (HU) settings. Bone window: window width (ww): 1,500 HU; window level (wl): 300 HU; brain window: ww: 100 HU; wl: 50 HU.

1. Planes of reference for orbital fracture (PROF):
PROF was a methodology previously developed to perform reproducible measurements of the orbit in orbital blowout fractures. 8 Four planes were identified on the CT scans: 8 Frankfurt plane 9 ( Fig. 1A ), midsagittal plane 9 ( Fig. 1B ), plane passing through the lowest point of the sella turcica ( Fig. 1C ), and the retroglobe plane of the unfractured orbit ( Fig. 1D ).
2. Midpoint of orbit:
At the retroglobe plane, the midorbital point of the unfractured orbit was obtained by identifying the midpoint of the medial and lateral most points of the orbit ( Fig. 1E ). The midorbital point, L4, was reflected across the midsagittal plane to identify the midorbital point of the fractured orbit ( Fig. 1F ).
3. Obtaining L1–7:
Viewed coronally, the distance between the medial and lateral most points of the unfractured orbit was measured and divided to obtain nine separate sagittal slices. Excluding the medial and lateral most points, we derived seven slices (L1–7), with L4 as the midorbital point. L1 (lateral-most) to L7 (medial-most) were plotted on the fractured orbit ( Fig. 1G ).

### Distance to Posterior Ledge

At L1–7, the distance between the anterosuperior-most point of IM and the anterosuperior-most point of the PL was measured ( Fig. 1H ). With measurements for the seven sagittal slices identified, we were able to plot the outline of the orbital floor fracture. Measurements were done in the bone window setting.

### Optic Foramen

The optic nerve is a critical structure to be avoided during dissection within the deep orbit. The location of the optic foramen in relation to L1–7 was determined on the fractured orbit.

On the sagittal view, a change in angle between the anterior cranial fossa and the sella turcica was used to determine an axial slice of interest ( Fig. 1I ). The position of the optic foramen on this axial slice was located with reference to L1–7 . The optic foramen was visualized at L7. Measurements were taken at L7 to determine the distance between the anterosuperior-most point of the sphenoid bone forming the optic foramen and the anterosuperior-most point of the IM ( Fig. 1J ).

## Results

### Demographic Data

The 67 patients' ages range from 21 to 69 years old (mean: 33.9). There are 14 (20.9%) women and 53 (79.1%) men. Thirty-one (46.3%) of the fractures are located in the left orbit, and 36 (53.7%) in the right. Assault caused the majority of the fractures (31.3%; Table 1 ).

**Table 1:** Demographics and etiology of trauma

Variable	Frequency (%) *N* = 67
Mean age (range)	37.8 (21–69)
Gender	
Female	14 (20.9)
Male	53 (79.1)
Side of fracture	
Left	31 (46.3%)
Right	36 (53.7%)
Mechanism of injury	
Assault	21 (31.3)
Sports related	15 (22.4)
Road traffic accident	12 (17.9)
Fall	8 (11.9)
Others	8 (11.9)
Unknown	3 (4.5)

### Distance of Posterior Ledge from Infraorbital Margin

**Fig. 2:**
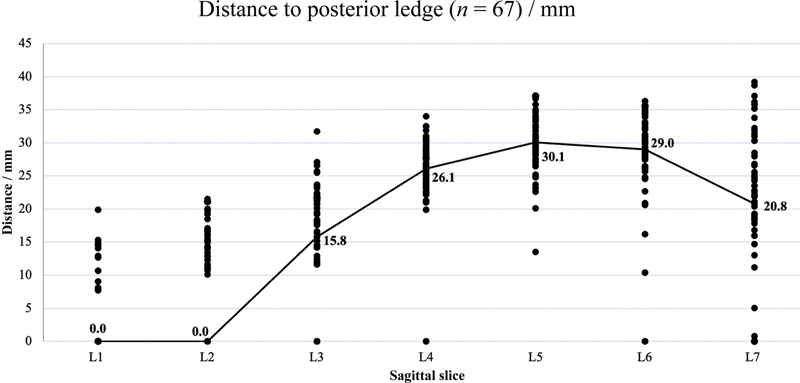
Scatter plot showing distance of posterior ledge from infraorbital margin for L1–7 with median distance represented by trendline.

The median distance of PL from IM increases medially until L5 before decreasing ( Table 2 ). An outline of the PL is plotted from these data ( Fig. 2 ).

**Table 2:** Median and range of distances of posterior ledge from infraorbital margin for L1–7

Slice	Median distance (mm)	Range (mm)
L1	0.0	0.0–19.9
L2	0.0	0.0–21.5
L3	15.8	0.0–31.7
L4	26.1	0.0–34.0
L5	30.1	13.5–37.1
L6	29.0	0.0–36.3
L7	20.8	0.0–39.2

### Location of the Optic Foramen

**Fig. 3:**
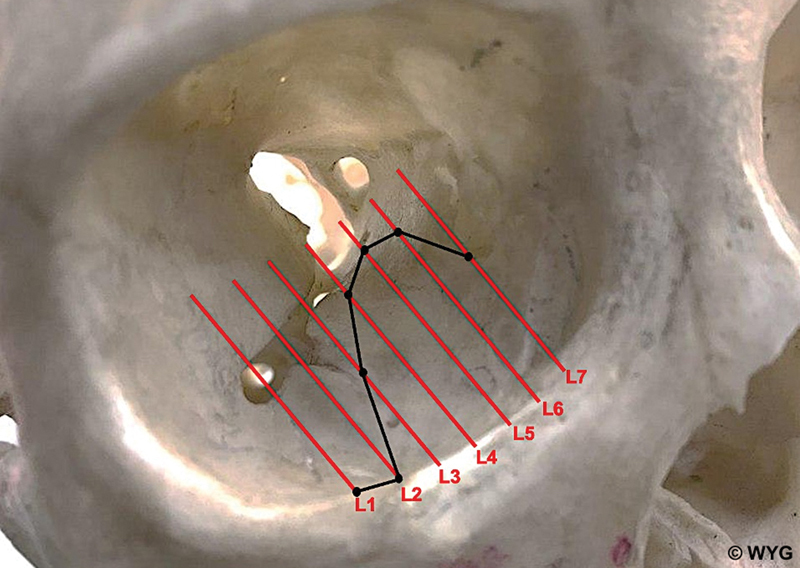
The location of the optic foramen and posterior ledge (PL) on an illustration of the skull. The optic foramen is located at L7. Solid line denotes PL. Mean distance between sagittal slices is 4.1 mm (range: 3.3–5.0).

The optic foramen is situated at L7 for 66 (98.5%) of the patients. The median distance of the optic foramen from IM is 43.7 mm (range: 37.0–49.1). The location of the optic foramen and PL is represented on an illustration of the skull ( Fig. 3 ).

## Discussion

PL and IM are stable bony structures left intact after orbital floor blowout fractures. 2 10 11 Orbital reconstruction plates are anchored to these landmarks during reconstruction. Orbital repairs resulting in a greater than 2-mm deviation from the original optic axis may lead to noticeable complications such as diplopia and enophthalmos. 2 Direct visualization of PL and IM aids implant placement intraoperatively. 12 Studies recommend that PL be identified using anatomical landmarks such as the orbital plate of the maxilla and inferior orbital fissure. 13 Visualization can be challenging due to the presence of soft tissue in the limited space. 13 Essig et al describes the effectiveness of computer-assisted preoperative planning and intraoperative navigation in accurate orbital reconstruction. 14 While three-dimensional reconstructions provide good spatial visualization of fractures, two-dimensional CT imaging is widely accessible and simple to use for preoperative planning. 15

Previous CT studies describe methods to measure the distance to PL preoperatively. 8 13 One such study by Manchio et al determined the maximum distance of PL from orbital apex in the coronal view by multiplying the number of CT slices between the two landmarks by the known CT image interval. 16 Our study maps the PL with CT imaging for better characterization of an orbital floor fracture in two dimensions, without the need for extensive intraoperative exposure. Given the three-dimensional nature of the orbit, our two-dimensional reference map should be correlated intraoperatively and not used in isolation.

The greatest distance of PL from IM is located at L5. Surgeons can determine the greatest defect length and mesh implant size at this slice. Anatomical studies identify that the thin lamina papyracea of the ethmoid bone, which forms part of the medial orbital floor, fractures easily. 11 While the rest of the orbital floor varies from 0.6 to 1.0 mm in thickness, the lamina papyracea of the ethmoid bone is less than 0.5 mm in thickness. 17 We postulate that changes in bony trabeculae at the confluence of the superior orbital fissure and inferior orbital fissure may contribute to the ease of fracture, resulting in a larger defect found at L5.

Identification of the optic foramen is essential to minimize damage to the optic nerve. 4 Previous cadaveric measurements determined the distances of the optic foramen in relation to other landmarks such as the posterior ethmoidal foramen and anterior lamina crest. 18 The CT imaging measurement tool employed in our study has a similar precision to physical calipers used in cadaveric studies (0.1 mm). 19 Our CT measurements have comparable precision to cadaveric measurements while avoiding the inaccuracies of bone and periorbital soft tissue desiccation in cadaveric dried skulls. 20 21 Compared with cadaveric specimens, CT images are readily available for large-scale anatomical studies to be conducted. Multiple preoperative CT techniques are developed for identification of the optic foramen. 4 Kim et al show that the optic foramen lies approximately 44.0 to 50.0 mm from IM. 11 Turvey and Golden reveal that the distance of optic foramen from IM is approximately 1 cm longer at the medial orbital rim compared with the lateral orbital rim. 22

Our study shows that the optic foramen is located at L7. Dissection along the medial orbital floor calls for greater caution to avoid iatrogenic injury to the optic nerve. A cadaveric study by Danko and Haug determined that distance between the soft tissue at the optic foramen and the inferior orbital rim ranges from 36.5 to 42.3 mm. 23

Our methodology can be applied to further map the medial and lateral walls of the orbit and the orbital roof. This serves as a foundation upon which we are developing an automated program with the School of Computing, National University of Singapore.

### Clinical Vignette

**Fig. 4:**
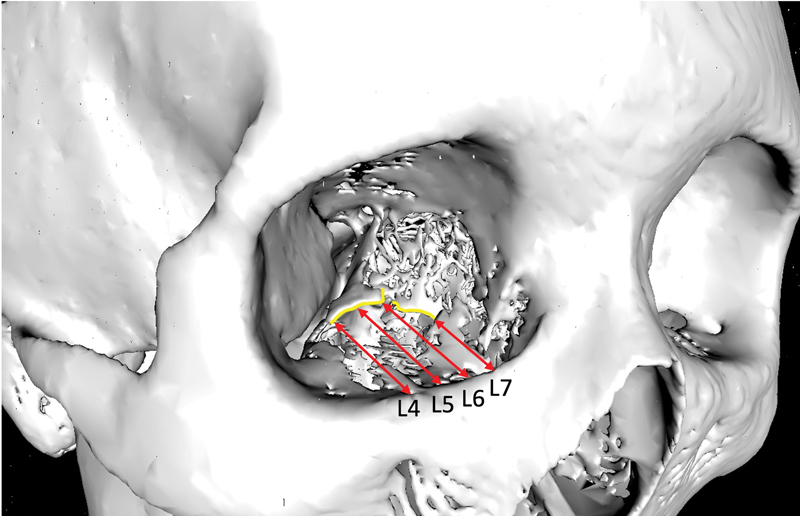
Three-dimensional reconstructed skull with L4–7 labelled showing distances (red arrows) between infraorbital margin and posterior ledge (yellow line).

**Fig. 5:**
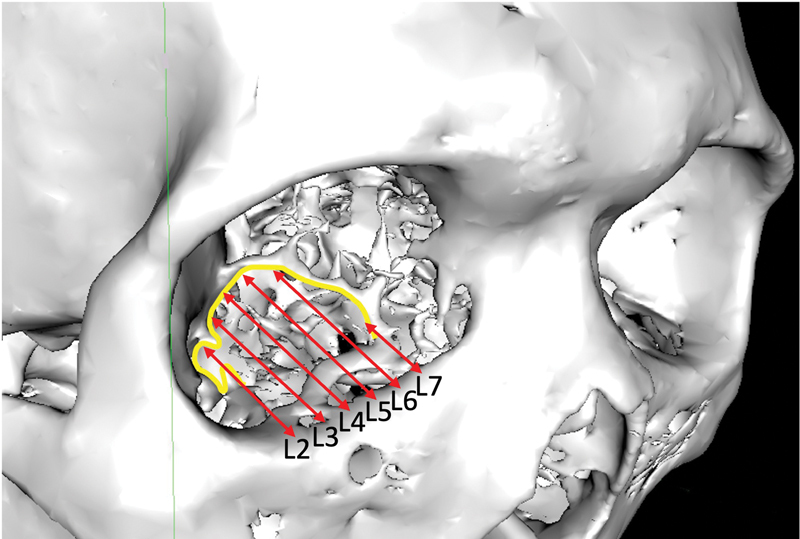
Three-dimensional reconstructed skull with L2–7 labelled showing distances (red arrows) between infraorbital margin and posterior ledge (yellow line).

1. A 27-year-old Chinese woman presents with a right orbital floor fracture after being assaulted. The fracture is visible from L4–7 ( Fig. 4 ). At L4, the distance of PL from IM is 23.3 mm. Distance of PL from IM increases to 27.5 mm at L5, before decreasing thereafter. The distance of the optic foramen from IM at L7 is 42.8 mm. A prefabricated titanium plate is used to reconstruct the orbital floor.
2. A 42-year-old Chinese man presents with a right orbital floor fracture after a sporting injury. The fracture is visible from L2–7 ( Fig. 5 ). The median distance of PL from IM increases medially from 15 mm at L2 to 30.4 mm at L5, before decreasing thereafter. A silastic implant is used to reconstruct the orbital floor.

This study presents a technique for generating a reference map of the orbital floor. This technique could be used to map fractures of other areas of the orbit for preoperative planning and anatomical studies. The distance of PL from IM increases medially until L5. The greatest defect is situated at L5. Caution should be exercised when performing deep orbital dissection medially.
